# Supplementary material for: An Oral Galectin Inhibitor in COVID-19—A Phase II Randomized Controlled Trial
Source: Vaccines (Basel). 2023 Mar 25;11(4):731. doi: 10.3390/vaccines11040731 (PMC10140888; doi:10.3390/vaccines11040731)
Supplement: Supplementary file 1 [file vaccines-11-00731-s001.zip › vaccines-2270784-supplementary.pdf]

Supplementary data:

Binding of PL-M to Gal-3 using HSQC NMR

Having established the in vitro and in vivo effectiveness of PL-M against SARS-CoV-2 infectivity [1,2], we sought insight on the molecular level. We hypothesized that PL-M functions in situ by binding to and antagonizing Gal-3, which is known to interact with SARS-CoV-2 to promote viral entry into cells [2]. To validate this proposal, we used NMR spectroscopy to assess binding interactions between PL-M and Gal-3, as well as between Gal-3 and SARS-CoV-2 S1 spike protein.

HSQC NMR spectra of ^{15}N-labeled Gal-3 (^{15}N-Gal-3) were measured as a function of PL-M concentration as reported in our previous manuscript [2] for ^{15}N-Gal-3 in the absence and presence of PL-M. Because resonances were broadened and minimally chemically shifted, we concluded that PL-M binding to Gal-3 falls in the inter-mediate exchange regime on the chemical shift time scale, suggesting that the equilibrium dissociation constant (KD) falls in the 2 μM to 100 μM range [3]. These NMR indicate that this broadening reflects binding avidity primarily because binding stoichiometry is un-known. Apparent broadening could also result from multiple binding modes given the heterogeneous nature of PL-M. In any event, these NMR demonstrated that Gal-3 binds relatively strongly to PL-M with the binding epitope on Gal-3 residing within Gal-3 CRD β-strands 3, 4, 5 and 6 that comprise the S-face β-sheet of the β-sandwich to which the β-galactoside lactose binds [2].

Binding of Gal-3 to SARS-CoV-2 S1 Spike Protein using HSQC NMR

To further validate our hypothesis, we used NMR spectroscopy in the present study to assess interactions between the viral spike protein S1 and Gal-3, both to the full-length lectin and to its truncated CRD form. HSQC NMR spectra of ^{15}N-labeled full length Gal-3 (Gal-3 FL) were measured as a function of spike protein concentration (0.2, 0.4, 1, 2, 4 and 10 μM). ^{15}N-^{1}H HSQC spectra are shown in Figure S1A for ^{15}N-Gal-3 FL in the absence (peaks in black) and presence (peaks in red) of 1 μM spike protein. As with PL-M binding, Gal-3 resonances are differentially chemically shifted and reduced in intensity (broadened), with some peaks becoming so broad by the end of the titration that they could not be observed, demonstrating that Gal-3 FL binding to the spike protein also occurs in the intermediate exchange regime on the chemical shift time scale with an apparent KD lying in the 2 μM to 100 μM range. Figure S1B shows that Gal-3 FL resonance broadening is about 50% at 1 μM spike protein, with effects within the NT and CRD occurring to the same ex-tent. This suggests that the NT itself, and not only the CRD, may play a role in binding to the spike protein.

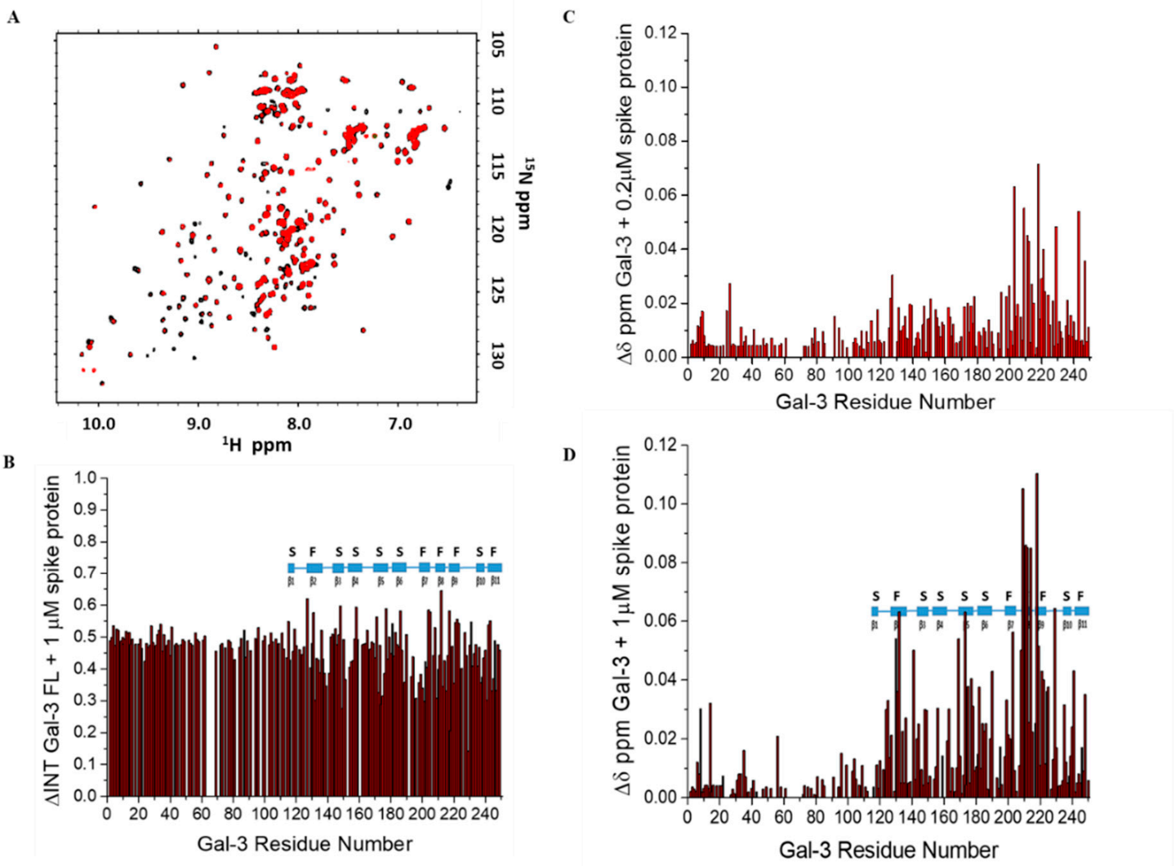

Figure S1. Gal-3 binding to SARS-CoV-2 spike protein.

Figure S1C plots ^{15}N-Gal-3 FL chemical shift changes, Δδ, vs. the amino acid sequence of Gal-3 at the spike protein concentration of 0.2 μM. Here, it is important to note that while canonical sugar binding S-face residues are chemically shifted, the most shifted resonances actually arise from Gal-3 residues within β-strands 2, 7, 8 and 9 that comprise the F-face β-sheet to which the NT transiently interacts [4]. This indicates that the initial interaction site with the spike protein may be on the F-face of the lectin. However, at 1 μM spike protein (Figure S1D), binding to the Gal-3 FL CRD S-face becomes more enhanced as one might expect.

HSQC spectra of ^{15}N-labeled truncated Gal-3 CRD were also measured as a function of spike protein concentration (0.2, 0.4, 1, 2, 4 and 10 μM). ^{15}N-^{1}H HSQC spectra are overlaid in Figure S2A for ^{15}N-Gal-3 in the absence (peaks in black) and presence (peaks in red) of 1 μM spike protein. Even though it appears that binding of both Gal-3 FL and truncated Gal-3 CRD to the spike protein occur to similar extents, as reflected in changes in resonance broadening (Figure S2B,C), Δδ values for truncated Gal-3 CRD at 1 μM spike protein are relatively very small and more scattered throughout the CRD, both S- and F-faces (Figure S2D), compared to Gal-3 FL. This suggests that Gal-3 FL with its N-terminal tail (NT) may provide an additional binding epitope for interactions with the spike protein. How-ever, when we used the truncated Gal-3 N-Terminal tail (NT, residues 1-113), spectral changes in the presence of spike protein are minimal. Therefore, we conclude that spike protein binding to the Gal-3 CRD S-face induces allosteric changes within the NT which is known to be in dynamic equilibrium with the Gal-3 CRD F-face [4].

Figure S2C plots ΔINT values averaged over all Gal-3 resonances for both Gal-3 FL and Gal-3 CRD vs. the concentration of the spike protein. In both cases, broadening plateaus out at ~4 μM spike protein, suggesting that binding is saturated at this concentration. Nevertheless, it is difficult to say that this is indeed the case, because binding interactions occur in the intermediary exchange regime where KD values cannot be accurately determined [3]. Nevertheless, if binding were to saturate at ~4 μM spike protein with Gal-3 at

20 μM, binding stoichiometry would be ~5 molecules of Gal-3 per 1 molecule of spike protein, and thus ~5 Gal-3 binding sites on the Omicron variant spike protein, a stoichiometry that seems reasonable.

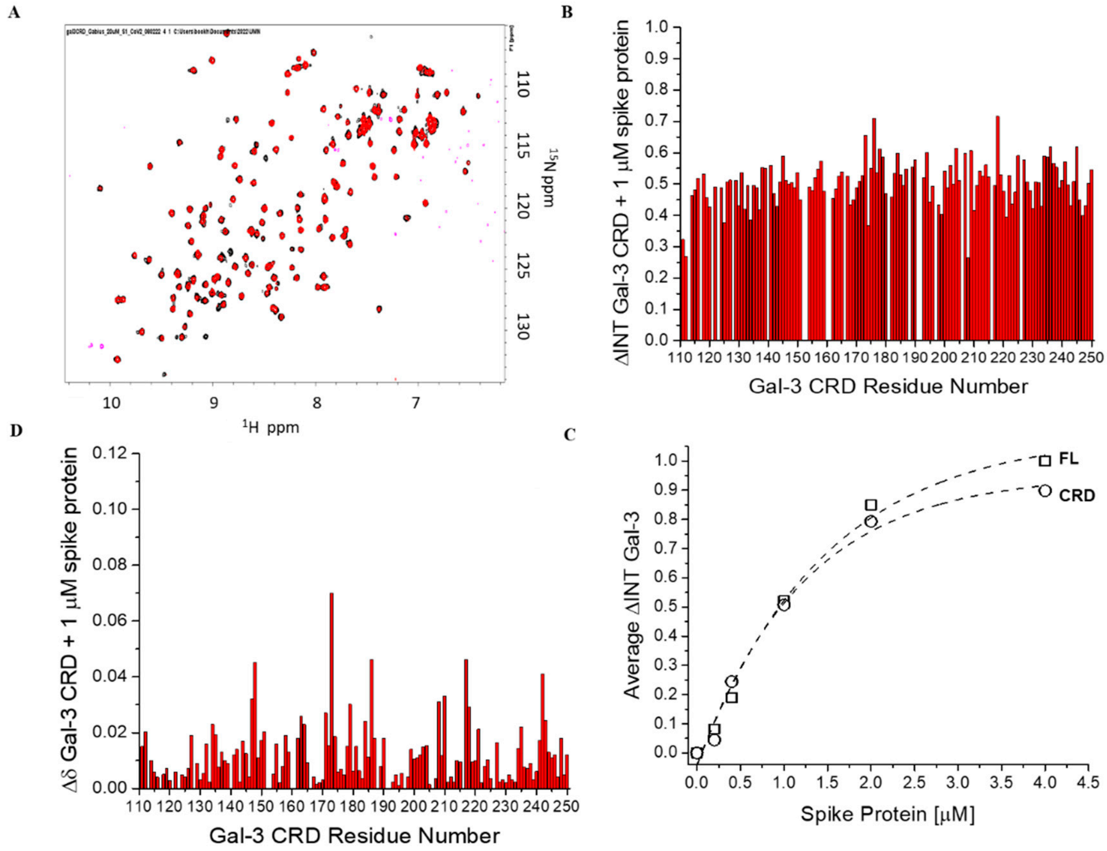

Figure S2. Interaction of Gal-3 to SARS-CoV-2 S1 Spike Protein using HSQC NMR spectroscopy.

Lactose competes with Gal-3 to SARS-CoV-2 Spike Protein

The β-galactoside lactose is the classical ligand that binds to the canonical sugar binding S-face of the galectin CRD. To assess whether the Gal-3 CRD binds to β-galactoside groups on the spike protein, we added lactose to the mixture of Gal-3 and spike protein to see if lactose could compete off Gal-3 binding to the spike protein. Here, we report that lactose indeed induces a reduction in Gal-3 resonance broadening, indicating a reduction in Gal-3 binding to the spike protein. In the absence of lactose, Gal-3 FL resonances are highly broadened, consistent with binding of Gal-3 FL to the SARS-CoV-2 S1 spike protein as discussed above (Figure S3A). Resonance broadening (ΔINT) averaged over all residues 1-250 is 0.911, whereas ΔINT averaged over residues 1-113 (NT) and 114-250 (CRD) are 0.816 and 0.972, respectively. Addition of 10 mM lactose greatly de-creases Gal-3 FL resonance broadening induced by its equilibrium binding to the spike protein (Figure S3B). In this instance, ΔINT values averaged over all residues 1-250, residues 1-113 and residues 114-250 are 0.473, 0.341, and 0.558, respectively, indicating that lactose competes with glycans on the surface of the spike protein for binding to Gal-3 FL. However, it is important to note that the lactose-induced reduction in Gal-3 FL resonance broadening occurs differentially, with some residues showing minimal if any change and others showing a large change (Figure S3B). This differential effect suggests that the Gal-3 glycan-binding epitope on the spike protein involves carbohydrates other than, and in addition to, β-galactosides. In fact, Lenza et al. reported that Gal-3 can bind to various glycosides on the spike protein [5].

With truncated Gal-3 CRD, resonances are also highly broadened in the presence of the spike protein (Figure S3C), with ΔINT averaged over residues 114-250 being 0.898. In

this instance, addition of 10 mM lactose greatly decreases Gal-3 CRD resonance broadening throughout the entire CRD (Figure S3D), with ΔINT averaged over residues 114-250 being 0.232. Thus, lactose more effectively competes off truncated Gal-3 CRD from the spike protein compared to Gal-3 FL. Moreover, it appears that lactose competes well with glycans on the surface of the spike protein for binding to truncated Gal-3 CRD, suggesting that the glycan binding profile for truncated Gal-3 CRD is different from that for Gal-3 FL.

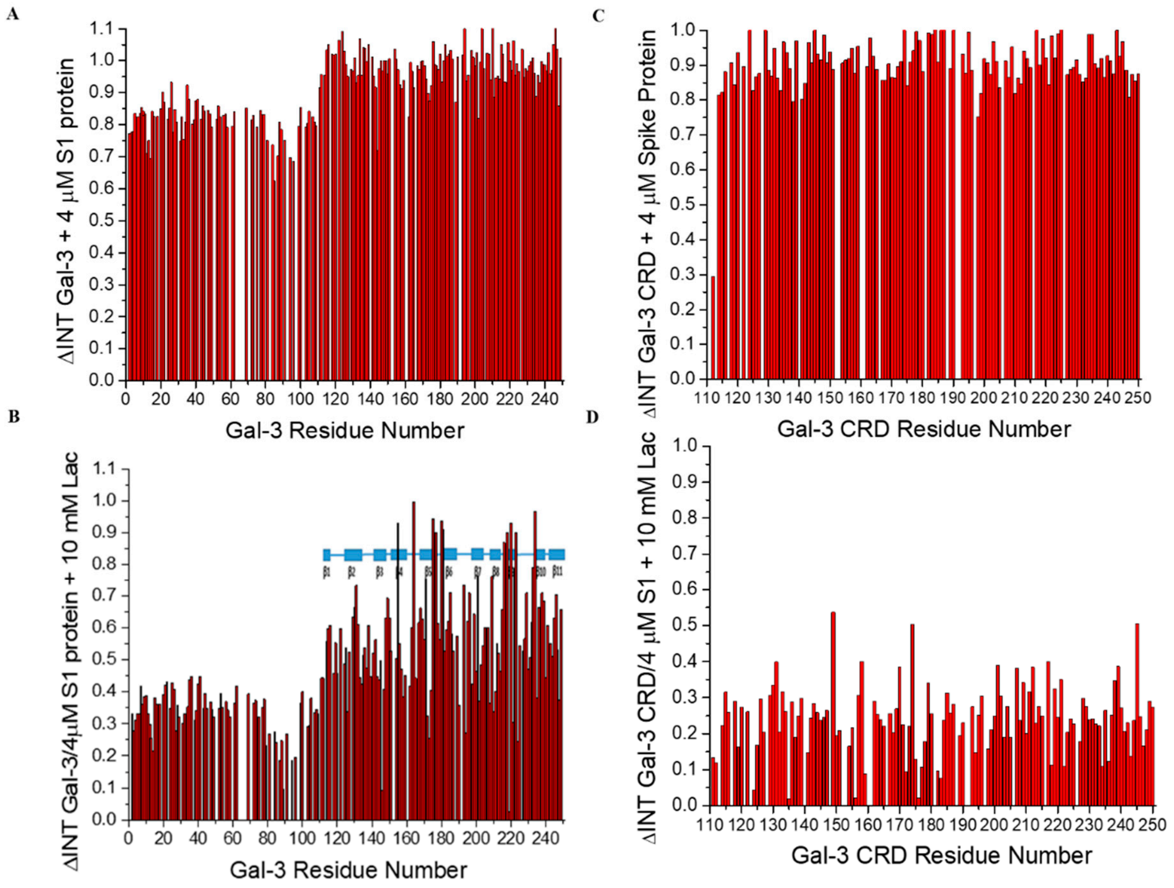

Figure S3. Lactose competes with Gal-3 to SARS-CoV-2 Spike Protein using HSQC NMR spectroscopy.

References

1. Sigamani, A.; Ruthra, M.; Sudhishma Shetty, S.; Madhavi Chugani, A.; Chen-Walden, H.; Platt, D.; Kutty, T. Galectin Antagonist use in Mild Cases of SARS-CoV-2: Pilot Feasibility Randomised Open Label Controlled Trial. J. Vaccines Vaccin. 2020, S10, 1000003.
2. Sigamani, A.; Chen-Walden, H.; Pahan, J.; Miller, M.C.; Mayo, K.H.; Platt, D. Carbohydrate prolectin-M, a galectin-3 antagonist, blocks SARS-CoV-2 activity. Int. J. Health Sci. 2022, 6, 6671–6683.
3. Williamson, M.P. Using chemical shift perturbation to characterise ligand binding. Prog. Nucl. Magn. Reson. Spectrosc. 2013, 73, 1-16.
4. Ippel, H.; Miller, M.C.; Vértesy, S.; Zheng, Y.; Cañada, F.J.; Suylen, D.; Umemoto, K.; Romanò, C.; Hackeng, T.; Tai, G.; et al. Intra- and intermolecular interactions of human galectin-3: assessment by full-assignment-based NMR. Glycobiology 2016, 26, 888-903.
5. Lenza, M.P.; Oyenarte, I.; Diercks, T.; Quintana, J.I.; Gimeno, A.; Coelho, H.; Diniz, A.; Peccati, F.; Delgado, S.; Bosch, A.; et al. Structural Characterization of N-Linked Glycans in the Receptor Binding Domain of the SARS-CoV- 2 Spike Protein and their Interactions with Human Lectins. Angew. Chem. Int. Ed. Engl. 2020, 59, 23763-23771.
